# Shape-directed self-assembly of nanodumbbells into superstructure polymorphs†

**DOI:** 10.1039/d0sc00592d

**Published:** 2020-03-27

**Authors:** Yulian Liu, Kerong Deng, Jun Yang, Xiaotong Wu, Xiaokun Fan, Min Tang, Zewei Quan

**Affiliations:** Department of Chemistry, Shenzhen Engineering Research Center for Frontier Materials Synthesis at High Pressures, Southern University of Science and Technology (SUSTech) Shenzhen Guangdong 518055 China quanzw@sustech.edu.cn; School of Chemistry and Chemical Engineering, Southwest University Chongqing 400715 China

## Abstract

Self-assembly of colloidal nanoparticles into ordered superstructures provides a promising route to create novel/enhanced functional materials. Much progress has been made in self-assembly of anisotropic nanoparticles, but the complexity and tunability of superstructures remain restricted by their available geometries. Here we report the controlled packing of nanodumbbells (NDs) with two spherical lobes connected by one rod-like middle bar into varied superstructure polymorphs. When assembled into two-dimensional (2D) monolayer assemblies, such NDs with specific shape parameters could form orientationally ordered degenerate crystals with a 6-fold symmetry, in which these NDs possess no translational order but three allowed orientations with a rotational symmetry of 120 degrees. Detailed analyses identify the distinct roles of subunits in the ND assembly: the spherical lobes direct NDs to closely assemble together into a hexagonal pattern, and the rod-like connection between the lobes endows NDs with this specific orientational order. Such intralayer assembly features are well maintained in the two-layer superstructures of NDs; however, the interlayer stackings could be adjusted to produce stable bilayer superstructures and a series of metastable moiré patterns. Moreover, in addition to horizontal alignment, these NDs could gradually stand up to form tilted or even vertical packing based on the delicate control over the liquid–liquid interface and ND dimensions. This study provides novel insights into creating superstructures by controlling geometric features of nanoscale building blocks and may spur their novel applications.

## Abstract 2

Nanodumbbells were assembled into superstructures with three orientations, and distinct roles of individual subunits of nanodumbbells in the assembly are uncovered.

## Graphical abstract

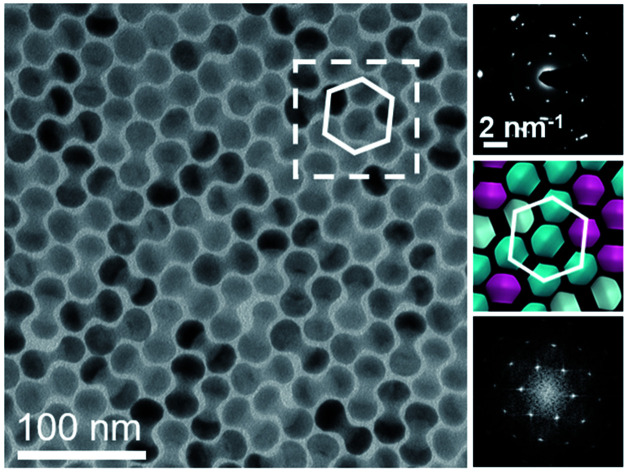

## Introduction

Self-assembly of nanoparticles (NPs) into ordered structures is an intriguing method for fabricating artificial materials and also facilitates the visualization and understanding of condensed phases.^1–12^ The use of spherical NPs as building blocks usually produces assembly superstructures that possess long range translational order but no orientational order. Non-spherical NPs with varied shapes have been used to design and produce ordered structures with spatial symmetries and anisotropic properties that are unattainable for spherical NPs. As for self-assembled superlattices consisting of simple non-spherical NPs such as nanocubes and nanooctahedra, they can be usually endowed with additional orientational order in their assemblies. Furthermore, the geometry features of NPs play a key role in determining their assembly behaviors, and a diverse and complex assortment of analogous superstructures such as crystals, plastic crystals and liquid crystals can be produced based on variously shaped NP building blocks.^13–26^ As a notable example, tetrahedra have been recently demonstrated to form various assemblies, including one-dimensional chiral tetrahelices, two-dimensional quasicrystals and three-dimensional cluster-based body-centred-cubic single supercrystals.^27–30^ Despite great efforts devoted to non-spherical NPs, systematic self-assembly investigations on anisotropic NPs with peculiar geometries are still rare.

Dumbbells geometrically consist of two lobes connected by a middle bar, which is the crudest model for NP dimers and the simplest nonconvex body. The enlarged heads with respect to the middle region in the dumbbells provide additional steric repulsions to restrict their assembly along certain directions, rendering them interesting building blocks for self-assembly investigations.^31–36^ Theoretical calculations predicted that the symmetric dumbbells can selectively induce the formation of orientationally disordered degenerate crystals, herringbone crystals and ordered oblique-lattice crystals.^33,37–40^ Experimental demonstrations have also been performed, including the parallel arrangement and cross-like dimers of gold NDs,^41,42^ the controlled orientations of NDs under external fields,^35,43–46^ and the self-assembly of magnetic asymmetric dumbbells into chainlike structures with tunable chirality.^45^ However, how to delicately control the self-assembly behaviors of dumbbells and obtain a series of novel assembly polymorphs with large-scale ordering remains poorly accessible.

Here we present the intriguing assembly behaviors of monodisperse core–shell β-NaYF_4_:Yb/Er@NaGdF_4_ NPs with a well-defined dumbbell morphology. These NDs can be controllably assembled into diverse assembly polymorphs, greatly expanding the structural complexity of NP assemblies. Intriguingly, these intralayer NDs possess no long-range translational order but one specific orientational order, that is, three allowed orientations with a rotational symmetry of 120 degrees. Further analyses indicate that the delicate control over ND shape parameters is essential for the formation of such orientationally ordered crystal. Furthermore, the horizontal orientations of interlayer NDs within two layers can be relatively adjusted to form either ordered thermodynamically stable 2D superstructures or kinetically trapped moiré patterns. Other tilted and perpendicular alignments of NDs could also be obtained by adjusting the subphase solvent and/or their shape parameters.

## Results and discussion

**Fig. 1:**
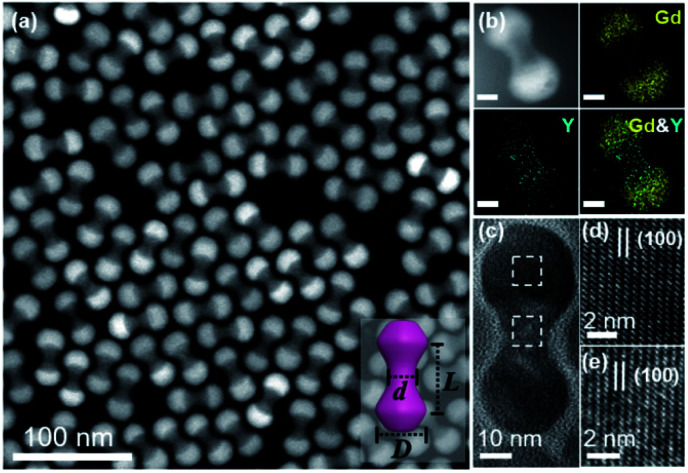
TEM characterization of core–shell β-NaYF_4_:Yb/Er@NaGdF_4_ NDs. (a) HAADF-STEM image of the as-synthesized core–shell NDs. Inset: schematic of a single ND with several geometric parameters: the distance between the center of two lobes (*L*), the diameter of the lobe (*D*) and the width of the middle part (*d*). (b) HAADF-STEM image and elemental mappings of a representative ND (*L*/*D* = 1.08, *D*/*d* = 2.08). Scale bar: 10 nm. (c) HR-TEM image of a representative ND. (d and e) HR-TEM images of the lobe and middle part marked with squares in (c), respectively.

Core–shell β-NaYF_4_:Yb/Er@NaGdF_4_ NCs were constructed *via* a reported protocol with certain modifications.^47^ In this process, an inert β-NaGdF_4_ shell was epitaxially grown outside the surface of β-NaYF_4_:Yb/Er ellipsoid core NPs (Fig. S1†). Due to the selective growth of the shell along the longitudinal direction in the presence of a high ratio of oleate anions (OA^−^)/oleate molecules (OAH), dumbbell-shaped NCs are formed. The high-angle annular dark-field scanning transmission electron microscopy (HAADF-STEM) image reveals the typical morphology of core–shell heterostructured NDs, which consist of two lobes (like spheres) connected by a rod-like central shaft (Fig. 1a). As demonstrated in the inset of Fig. 1a, the shape of the symmetric dumbbell is described by two values (*L*/*D* and *D*/*d*), where *L* is the distance between the centers of two lobes, *D* is the diameter of the lobe and *d* is the width of the middle bar. Besides these NDs with a size aspect ratio (*L*/*D*) of 1.08 (Fig. 1), different aspect ratios (1.07, 1.18 and 1.48) of core–shell NDs could be obtained by simply adjusting the feeding amount of NaOH/NH_4_F during the syntheses (Fig. S2 and Table S1†), and the details can be seen in the Experimental section. Combined with elemental mapping analyses (Fig. 1b), the bright and dark contrasts in the ends and the middle part of the ND originate from the presence of the heavier gadolinium element in the NaGdF_4_ shell and the lighter yttrium element in the NaYF_4_ core, respectively. As marked in the X-ray diffraction (XRD) results (Fig. S3†), the presence of a broad peak around 30 degrees also suggests the coexistence of β-NaYF_4_:Yb/Er and β-NaGdF_4_. High-resolution TEM (HR-TEM) images reveal the inter-planar spacings of 5.38 Å (Fig. 1d) and 5.29 Å (Fig. 1e), corresponding to the {100} planes of the NaGdF_4_ shell and NaYF_4_ core, respectively. This small lattice mismatch is essential to form heterostructured core–shell NDs, in which their {100} planes consecutively go through the whole nanoparticle.^48–50^ The synthesized core–shell NDs are dispersed in hexane with a concentration of 14.5 mg mL^−1^ for the assembly use.

**Fig. 2:**
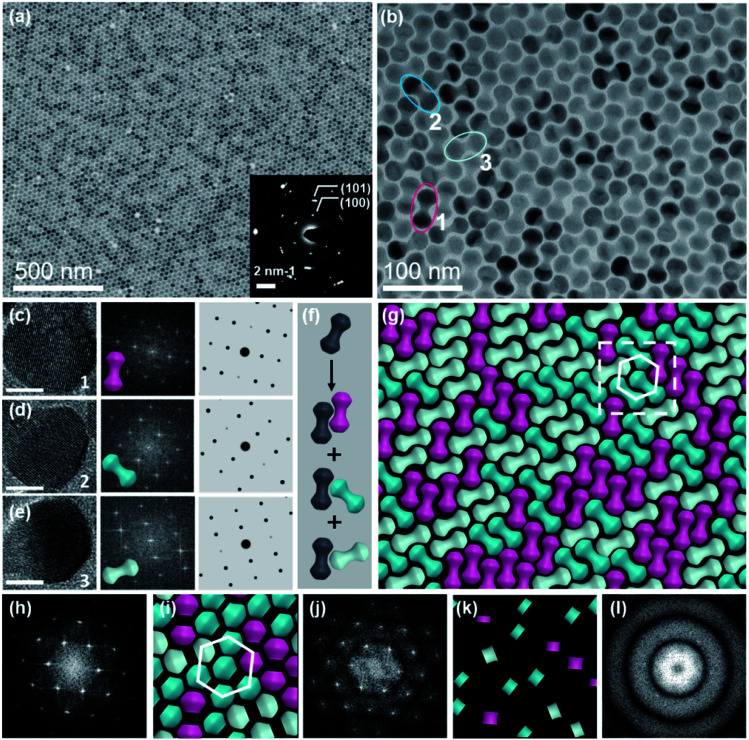
Large-scale monolayer membrane self-assembled from NDs on the EG subphase. (a) Representative TEM image of the ND monolayer membrane, with the lower inset showing the corresponding SAED pattern. (b) TEM image at a higher magnification. (c–e) HR-TEM images (left) of NDs with different orientations captured from (b), scale bar: 10 nm; corresponding FFT patterns (middle); and simulated FFT patterns (right). (f) Schematic of packing of NDs into three possible different bi-dumbbell structures. (g) Schematic of the monolayer membrane captured from (b). (h) FFT pattern of the monolayer TEM image (b). (i and k) Schematics of lobes and middle parts of NDs as marked in (g), respectively. (j and l) FFT patterns of lobes and middle parts of NDs obtained from (i and k), respectively.

In this system, all self-assembly experiments were performed at the liquid–air interface, as briefly described below. The NDs were assembled on the surface of ethylene glycol (EG) in a Teflon well at room temperature without disturbance (Fig. S4†). A glass slide was used to cover it to slow down the assembly process, and it usually takes 3–4 hours for the upper colloidal suspension of NDs to completely evaporate. Then, a thin film could be visible on the EG surface, and a substrate (*e.g.*, carbon-coated TEM grid or Si wafer) was used to transfer the membrane for further characterization. The large-scale monolayer membrane was prepared by evaporating a mixed solution consisting of 20 μL of the ND hexane stock solution (14.5 mg mL^−1^) and 60 μL of toluene on the EG surface. Fig. 2a shows a typical TEM image of the monolayer membrane, indicating that these NDs form 2D close-packed assemblies (Fig. S5†). The domain size of this monolayer assembly is estimated to be at least 50 μm from Fig. S6a.† It should be noted that the solvent composition plays a critical role in the formation of ordered ND assemblies. Without adding a toluene co-solvent into the stock solution, NDs prefer to be randomly packed together (Fig. S7†). As known, toluene possesses a higher boiling-point, and thus the evaporation rate of the mixed solvents could be obviously slowed down, which favors the formation of ordered ND packing. In addition, different types of solvents could directly affect the solvent–ligand interactions during the ND assembly process, as previously observed in other NP systems.^25,51,52^

These NDs seem to assemble together with a hexagonal array (Fig. 2a) that is the same as the 2D packing of equal spheres, while three ND orientations can be identified in the high-magnified TEM image (Fig. 2b). The corresponding selected-area electron diffraction (SAED) pattern in the inset of Fig. 2a indicates that NDs are not randomly aligned and there is a strong correlation between these ND orientations, as confirmed by the fast Fourier transform (FFT) pattern of the corresponding SEM image (Fig. S8†). To identify the orientations of NDs, HR-TEM images of the lobes of three representative NDs are obtained, accompanied by their corresponding experimental and theoretical FFT patterns, shown in Fig. 2c–e. The combined image of these three FFT patterns is consistent with their SAED pattern (inset of Fig. 2a), and thus these three orientations are revealed to have a relative rotation angle of 120 degrees. More details about the combination of the three simulated FFT patterns are shown in Fig. S9.†

For the ease of following structural analyses, the NDs are modeled with three colors to differentiate these orientations. As shown in Fig. 2g, such models are used to reconstruct the monolayer assembly, from which some underlying rules about this intriguing shape-directed self-assembly can be accordingly obtained. First, there are three kinds of ways for neighboring NDs to contact each other: parallel or at the fixed rotation angles of 60 degrees or 120 degrees (Fig. 2f). Meanwhile, the lobe position of one ND is always restricted to the middle section of the neighboring particle to achieve horizontal close-packing. Second, the spherical lobes and rod-like middle part are revealed to play distinct roles in the formation of this 2D assembly pattern. To identify their individual geometrical contributions, FFT analyses of separated lobes and middle parts in this constructed pattern are performed, as shown in Fig. 2i–l and S11–12.† Based on the schematic of ND lobes alone (Fig. 2i), a characteristic hexagonal symmetry is observed in the FFT pattern (Fig. 2j). This pattern matches well with the one (Fig. 2h) obtained from the TEM image of the ND monolayer assembly, indicating that the spherical lobes should be responsible for the hexagonal symmetry of ND assemblies. In contrast, no obvious spots are observed in the FFT pattern (Fig. 2l) of the middle parts of NDs (Fig. 2k), suggesting that middle parts alone are disordered. In another respect, in the 2D close-packed assembly of spheres, if the nearest two spheres are connected with lines (resembling one dumbbell), only three orientations with the rotation angle of 120 degrees can be obtained (Fig. S13†). This in turn confirms the key effect of the rod-like connection between two lobes on three allowed orientations of NDs in the 2D monolayer assembly. As mentioned above, the peculiar geometry endows NDs with such special packing ability. Interestingly, these NDs in this 2D monolayer assembly have three allowed orientations (one special orientational order); however, as a whole, they do not have translational order. The spherical lobes of NDs in this monolayer assembly not only possess translational order, but also are restricted to have three orientations, which is in contrast to the random orientations of spherical NPs in traditional hexagonal packing.

The formation of such close-packed superstructures can be explained from the aspect of energy minimization. It has been theoretically verified that the symmetric dumbbell shows a large configurational degeneracy in its close-packed structure.^37,53,54^ As demonstrated, such a high degeneracy entropy helps stabilize the aperiodic phase, in which the dumbbell arrangements can be represented by three orientations with apparent randomness.^38,55^ There is a substantial positive entropy contribution from the aperiodic phase, rendering the aperiodic structure more stable due to the negative contribution to the free energy.^55^ Further simulations indeed reveal that this aperiodic phase is thermodynamically stable in the whole range of solid density, when the shape parameter *L*/*D* is fixed approximately close to unity as discussed in this work.^37^ Such a phase is previously referred to as a degenerate crystal, in which orientations of NPs are not quite random. In this dumbbell system, the ND orientation populates one of the three crystalline directions with equal probability based on the statistical data analyses (Fig. S10†).

In another respect, the anisotropic parameters of NDs play a key role in determining their assembly structures.^32,39,56^ As shown in previous studies, a degenerate crystal can be produced as the two lobes in dumbbells are tangent or slightly overlapped.^39^ However, there are few reports about the self-assembly behaviors of elongated dumbbells, in which two lobes are separated and connected by a middle bar. For this purpose, NDs with varied shape parameters were assembled with the same method described above (Fig. S14†). As the middle bar length parameter (*L*/*D*) of NDs is larger than unity, the effect of the concave parameter (*D*/*d*) on the assembly behavior of NDs should be also taken into account. The width of the middle part (*d*) should be obviously smaller than the diameter of the lobe (*D*) to maintain its concave geometry; otherwise these NDs will behave like 1D nanorods to have the side-by-side arrangements (Fig. S14b†). In this system, limiting the shape parameters of NDs to *L*/*D* = 1.08 and *D*/*d* > 1 could produce an orientationally ordered degenerate superstructure (Fig. 2 and S14a†). Meanwhile, the geometric parameters should also satisfy the relationship of 0 ≤ *L* − *D* ≤ 2 nm (2 nm is the ligand length), to achieve the hexagonal array of spherical lobes. In the case of *L*/*D* > 1.08 and *L* − *D* > 2, orientationally disordered assemblies will be produced, as demonstrated in Fig. S14c and d.† Moreover, if the shape parameter of NDs is obviously reduced to smaller than unity such as *L*/*D* = 0.8 (Fig. S15†), orientationally disordered superstructure will be produced.

**Fig. 3:**
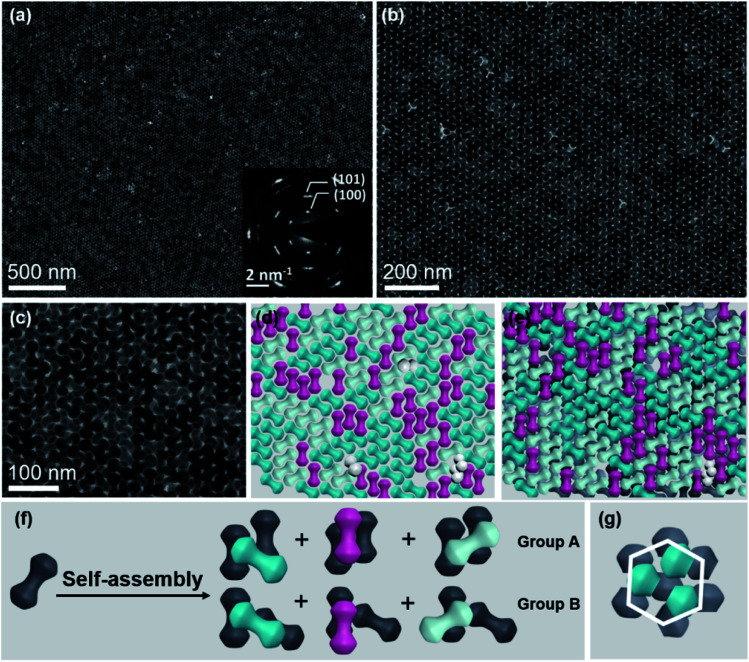
Large-scale bilayer membrane self-assembled on the EG subphase. (a–c) TEM images of the bilayer membrane at different magnifications. The inset of (a) shows the corresponding SAED pattern. (d) Schematic of NDs in the first layer captured from (c). Some cracks in this layer are filled with tilted NDs (white color). (e) Schematic of NDs in the double layer captured from (c), in which the gray color is used to represent NDs in the first layer. (f) Schematic of self-assembly of NDs into six different bilayer structures. (g) Hexagonal packing of ND lobes in this bilayer structure.

In addition to the monolayer assembly, these NDs are capable of forming an ordered bilayer structure, by increasing the volume of stock solution from 20 μL to 35 μL in the self-assembly process while keeping all other variables the same. Fig. 3a–c show the TEM images at different magnifications, from which it can be observed that these NDs seem to be well packed in a large scale. The intense spots in the FFT patterns calculated from their TEM and SEM images further confirm the ordered alignments of NDs in this bilayer structure (Fig. S16 and 17†). Similar to the monolayer assembly, the SAED pattern of this ND bilayer structure also displays a 6-fold symmetry (inset of Fig. 3a), indicating that the NDs in both layers should have the same set of three allowed orientations. To intuitively uncover the interlayer stacking mode, the schematics of separated bottom and top layers are extracted from the TEM image (Fig. 3c), as shown in Fig. 3d, e and S18,† respectively. This separation is based on the intralayer packing rules of NDs with three allowed orientations and the corresponding SAED and FFT patterns, and therefore the neighboring NDs of one ND within the same layer can be accordingly distinguished. It is easy to conclude that the NDs in the bottom and top layers have the same set of three allowed horizontal orientations (Fig. 3d and e). Further analyses reveal the vertical stacking manners of NDs between these two layers, as illustrated in Fig. 3f. As two NDs in the first layer are parallel (Group A) or aligned at a rotation angle of 120 degrees (Group B), the ND in the second layer is always either parallel to one of them or horizontally rotated by 120 degrees. This arrangement leads to the identical orientations of NDs in these two layers. Furthermore, the shape complementarity of convex lobes and concave rod-like connections of NDs promises formation of such stable structures.^16,57,58^ One common feature of these interlayer stackings is that the lobes and rod-like connections of NDs in the second layer always sit on the complementary sections in the first layer, respectively, to obtain a flat assembly surface (Fig. S16†).

To determine the individual geometrical contributions, similar FFT analyses of this bilayer structure are performed (Fig. S19 and 20†). The spherical lobes of NDs are hexagonally arrayed within each layer to have an AB stacking type (Fig. 3g), determining the close-packing behavior of NDs. The rod-like connection between the lobes endows NDs with this specific orientational order in both layers. Such packing behaviors can be furthermore demonstrated by the 3D packing modes of classical spheres (Fig. S21†), from which only three types of connections (or orientations) between two spheres can be observed in each layer.

**Fig. 4:**
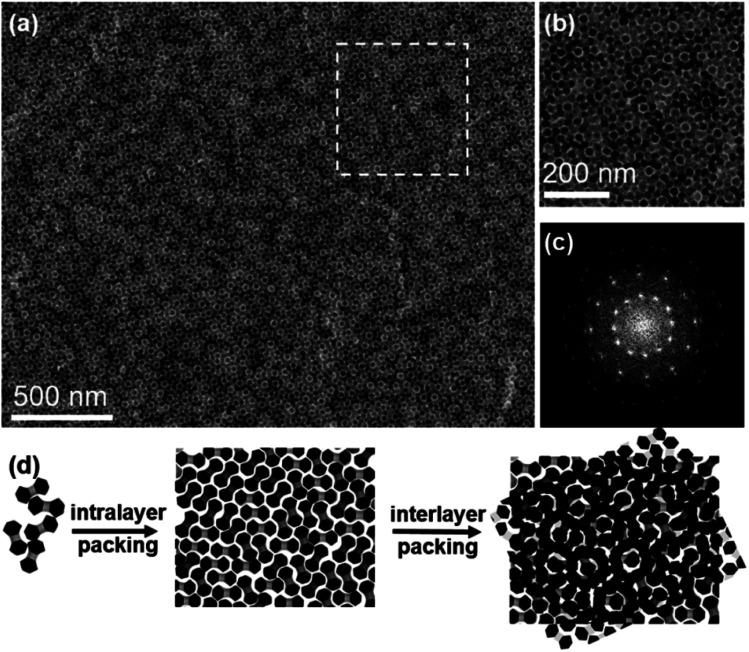
Quasi-12-fold moiré pattern generated from bilayer stacking of NDs on the EG subphase. (a) Representative TEM image of the quasi-12-fold moiré pattern. (b) TEM image at higher magnification. (c) Corresponding FFT pattern of (b). (d) Schematic demonstration of the formation of the quasi-12-fold moiré pattern based on the stacking of two ND monolayers.

The relative orientations of NDs in these two layers could be further adjusted to produce other metastable bilayer structures. By continuously increasing the volume of stock solution to 50 μL while keeping other assembly conditions the same, a series of intriguing moiré patterns (Fig. 4a and S22a†), which are strictly forbidden for periodic crystals, are obtained.^59,60^ Characterization of these moiré patterns in real and reciprocal spaces is performed to validate the assembly patterns of NDs. The FFT pattern of the magnified bilayer assembly structure (Fig. 4b) is shown in Fig. 4c, from which the rotation angles between two neighboring spots are measured to be 30 degrees. Thus, this moiré pattern possesses a typical 12-fold rotational symmetry.^61^ The presence of intense spots in the corresponding SAED pattern (Fig. S22a†) reveals that the NDs are not randomly packed together. Moreover, this SAED pattern can be regarded as the combination of two SAED patterns with a rotation angle of 30 degrees, which indicates the relative orientation of NDs in these two layers.^62^ As illustrated in Fig. 4d, the TEM image of this moiré pattern is reconstructed with the similar method used above for a regular bilayer assembly structure. It turns out that this moiré pattern can be produced by rotating two ND monolayers by 30 degrees, and NDs in each layer possess the same set of three allowed orientations.

The formation of this moiré pattern can be attributed to the misorientation of two ND monolayers, and thus the resulting moiré pattern should be aperiodic.^61^ Besides this dominant moiré pattern with a 12-fold symmetry, other moiré patterns with different rotation angles between two layers are also observed. For example, the rotation angle is determined to be 18 degrees for another type of moiré pattern, as shown in Fig. S22b, S23, and S24.† Compared with a regular bilayer structure, the NDs in these moiré patterns show three additional orientations, while the lobes of NDs still have no translational order. These moiré patterns based on anisotropic ND assembly are similar to those in traditional sphere assembly with varied misorientation angles.^59,63,64^ This feature in turn proves the key role of the spherical geometry feature of lobes in these NDs in the formation of these moiré patterns. The formation of these intriguing metastable superstructures is due to the kinetic trapping effect that frustrates the assembly process at a high ND concentration. In this system, such NDs at a lower concentration tend to form thermodynamically stable ordered monolayer and bilayer superstructures (Fig. 1 and 2), as there is sufficient time to allow these NDs to escape from the nonequilibrium state.^65,66^ As more NDs are present in a given volume of solvent, NDs have limited space to freely move, resulting in strong ND interactions. Consequently, NDs can be kinetically trapped before reaching equilibrium to form these metastable moiré patterns.^67^

**Fig. 5:**
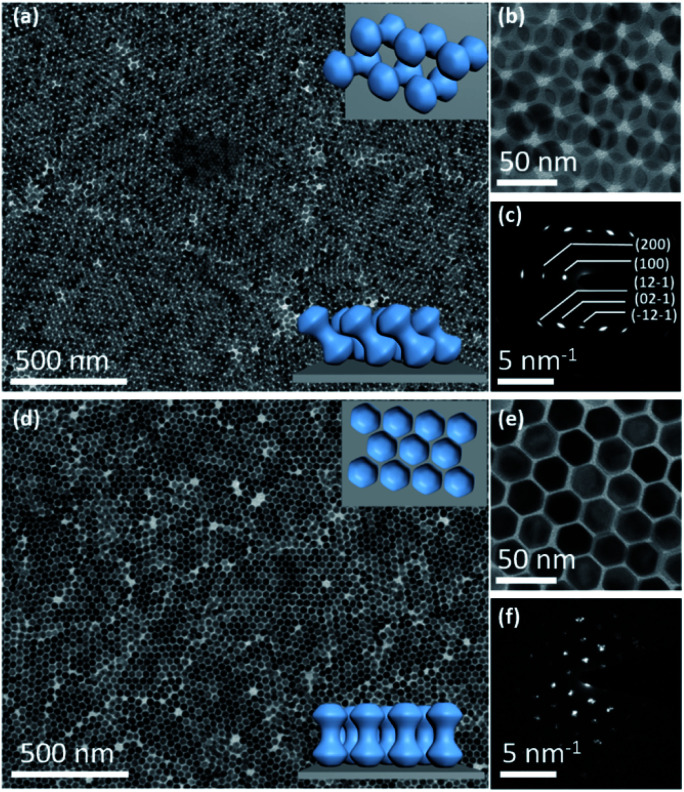
Self-assembly of NDs with different size aspect ratios (*L*/*D*) on the DMSO subphase. (a and b) Representative TEM images of tilted NDs (*L*/*D* = 1.08, *D*/*d* = 2.08) at different magnifications. Upper and lower insets of (a) show the schematics of tilted NDs from the top and front views, respectively. (c) Corresponding SAED pattern of (a). (d and e) Representative TEM images of vertically aligned NDs (*L*/*D* = 1.18, *D*/*d* = 1.87) at different magnifications. Upper and lower insets of (a) show the schematics of vertically aligned NDs from the top and front views, respectively. (f) Corresponding SAED pattern of (d).

Depending on the kind of subphase, the ND orientations relative to the horizontal liquid surface can be further adjusted from parallel in above assemblies to tilted and vertical. In the case of the DMSO subphase, these assembly domains display distinct assembly patterns (Fig. 5a and b), from which NDs possess a strong orientational order based on the SAED pattern (Fig. 5c). Combined with the corresponding SEM images (Fig. S25†), most NDs are demonstrated to stand slantwise on the substrate. The tilt angle is calculated to be about 36 degrees (Fig. S26†). The upper and lower insets of Fig. 5a show the schematics of tilted NDs from the top and front views, respectively. As described above, mixed hexane and toluene solvents are used to disperse these NDs to form colloidal suspensions, among which toluene is immiscible with EG but miscible with DMSO. In this case, the toluene solvent is readily diffused into bottom DMSO to become part of the subphase, and the NDs are actually dispersed in hexane for the following assembly process. Thus, similar tilted ND assemblies can also be observed when only hexane is used as the ND dispersion medium (Fig. S27†). The formation of tilted ND arrangement is attributed to the different molecular structures of EG and DMSO. Compared with EG that can form a strong hydrogen bond with a capping ligand molecule (oleic acid), DMSO has a much weaker capability in this respect.^68,69^ As a result, the NDs tend to lay on the EG subphase with their side surfaces that possess more oleic acid molecules than the head parts, to produce the close-packing patterns of horizontal NDs, as discussed above (Fig. 1–4). In contrast, the hydrogen bonding between DMSO and the oleic acid molecule is weak, and thus oleic acid molecules prefer to interact with each other to form the side-by-side packing of NDs. With a relatively big interparticle distance, the formation of ND assemblies with a slightly tilted orientation on the substrate is expected.

Moreover, the ND assembly is also sensitive to the dimensions of NDs on the DMSO subphase. By changing the size aspect ratio of NDs to *D*/*d* = 1.87, they can stand vertically on the substrate. Fig. 5d shows the large-scale film of vertically aligned NDs (*D*/*d* = 1.87) with a typical 2D hexagonal packing, confirmed with a magnified TEM image (Fig. 5e) and the corresponding SAED pattern (Fig. 5f), respectively. While NDs with a higher aspect ratio of *D*/*d* = 2.08 would provide more room (concavity) for accommodating its lobes to form a tilted arranged array, NDs with a smaller aspect ratio of 1.87 have a thicker middle connection between two lobes, making the shape behave like nanorods.^70^ Therefore, these NDs tend to stand vertically to have side-by-side assemblies on the subphase.^71–73^ This study further highlights the important role of the subphase and size aspect ratio of NDs in the self-assembly process.^74^

## Conclusions

In summary, this study has reported the self-assembly of anisotropic dumbbells into a series of 2D superstructure polymorphs including close-packed monolayer films with intralayer NDs displaying no translational order but three allowed orientations, and double-layer superstructures with controllable interlayer packing. The pairing constraints about the orientation and position of NDs in this assembly pattern are expected to produce intriguing mechanical properties such as a low Poisson ratio, which would enhance their piezoelectric properties. Besides the horizontal alignment, these NDs could gradually stand up to form tilted or even vertical packing through delicate control over the liquid–liquid interface and their geometrical dimensions. This shape directed self-assembly of NDs not only expands the diversity of superstructures but also provides another way to design new complex nanoassemblies.

## Experimental

### Materials

Rare earth chlorate hydrates (99.9%), oleic acid (OA) (90%), 1-octadecene (ODE) (90%) and NaOH (>97%) were purchased from Sigma-Aldrich (USA). NH_4_F (AR), ethanol, hexane, toluene, ethylene glycol (EG) (>90%) and dimethylsulfoxide (DMSO) (>99.8%) were received from Aladdin. All chemicals were used without any purification.

### Synthesis of core β-NaYF_4_:18%Yb^3+^,2%Er^3+^ NPs

Core NaYF_4_:18%Yb^3+^,2%Er^3+^ NPs were synthesized through a previously reported method with certain modifications. Generally, 1 mmol ReCl_3_·6H_2_O (Re = 80% Y, 18% Yb, 2% Er), 6 mL of OA and 15 mL of ODE were added into a 100 mL three-necked round-bottom flask. The mixture was heated to 150 °C under vigorous stirring to form a transparent solution. After being cooled down to room temperature, 6 mL of methanol solution containing NH_4_F (4 mmol) and NaOH (2.5 mmol) was injected to the flask. Subsequently, the solution was heated to 110 °C and degassed under vacuum for 30 minutes with constant stirring. Then the flask was refilled with argon and heated to 300 °C in 12 min, and kept for 1 h under vigorous stirring. After the mixture was cooled down to room temperature, the NPs were washed with 60 mL of ethanol and separated by centrifugation (4500 rpm, 4 min). After being washed twice, the NPs were dispersed in 5 ml hexane for further use.

### Synthesis of core–shell β-NaYF_4_:18%Yb,2%Er@NaGdF_4_ NDs

The preparation of the core–shell β-NaYF_4_:18%Yb,2%Er@NaGdF_4_ NDs is similar to that of the core NPs. Shell precursor solutions were prepared by heating GdCl_3_·6H_2_O (0.4 mmol), 6 mL of OA and 16 mL of ODE in a 50 mL three-neck round-bottom flask at 150 °C for 30 min. After being cooled down to room temperature, 1.4 mL of core NPs were added, followed by a methanol solution containing NH_4_F (1.4 mmol) and NaOH (4.3 mmol). The mixture was subsequently heated to 80 °C and kept for 10 min before it was heated to 110 °C for degassing under vacuum. After 30 min, the system was heated to 310 °C under an argon atmosphere and kept for about 1 h. The NDs were precipitated with 60 mL ethanol and separated by centrifugation at a speed of 4500 rpm for 4 min. This process was repeated twice. Finally, the NDs were dispersed in hexane for further use with a concentration of 14.5 mg mL^−1^.

### Self-assembly of NDs into superstructure polymorphs

The controlled self-assembly of NDs was achieved through a solvent evaporation process at the liquid–air interface. In a typical assembly process, 20 μL of stock solution containing core–shell β-NaYF_4_:18%Yb,2%Er@NaGdF_4_ NDs with a concentration of 14.5 mg mL^−1^ was mixed with 60 μL of toluene under ultrasonication for 2 s. The mixed colloidal suspension was evaporated on the EG subphase in a Teflon well, which was immediately covered with a transparent glass slide for observing the variations during the evaporation process. After the assembly process, a solid membrane was visible on the subphase. The membrane was transferred to a carbon supported TEM grid and Si wafer for further characterization.

### Characterization

X-ray diffraction (XRD) characterization was carried out using an X-ray diffractometer (Rigaku SmartLab) equipped with Cu Kα radiation (*λ* = 1.54 Å). Transmission electron microscopy (TEM) images were obtained on a Hitachi 7700 transmission electron microscope at 100 kV. High-resolution TEM (HR-TEM) and selected area electron diffraction (SAED) images were recorded on a FEI Tecnai F30 TEM with an accelerating voltage of 300 kV. High-angle annular dark field scanning TEM (HAAD-STEM) analyses and elemental mapping were conducted on a FEI Talos 200X operated at an accelerating voltage of 200 kV. Scanning electron microscope (SEM) images were captured on a field-emission SEM (FE-SEM, ZEISS Merlin) at 3 kV. The sizes of NPs were analyzed from TEM images using Nano Measurer software at least 100 particles per sample.

## Conflicts of interest

There are no conflicts of interest to declare.

## Supplementary Material

SC-011-D0SC00592D-s001
