# Supplementary material for: Shape-directed self-assembly of nanodumbbells into superstructure polymorphs
Source: Chem Sci. 2020 Mar 27;11(16):4065–73. doi: 10.1039/d0sc00592d (PMC8152806; doi:10.1039/d0sc00592d)
Supplement: SC-011-D0SC00592D-s001 [file SC-011-D0SC00592D-s001.pdf]

Electronic Supplementary Material (ESI) for Chemical Science.
This journal is © The Royal Society of Chemistry 2020

Supporting Information for

Shape-Directed Self-Assembly of Nanodumbbells into Superstructure Polymorphs

Yulian Liu,[1,2,#] Kerong Deng,[1,#] Jun Yang,[2] Xiaotong Wu,[1] Xiaokun Fan,[1] Min Tang,[1] and Zewei Quan[1,*]

1 Department of Chemistry, Shenzhen Engineering Research Center for Frontier Materials Synthesis at High Pressures, Southern University of Science and Technology (SUSTech), Shenzhen, Guangdong 518055, China

2 School of Chemistry and Chemical Engineering, Southwest University, Chongqing 400715, China

# equal contribution

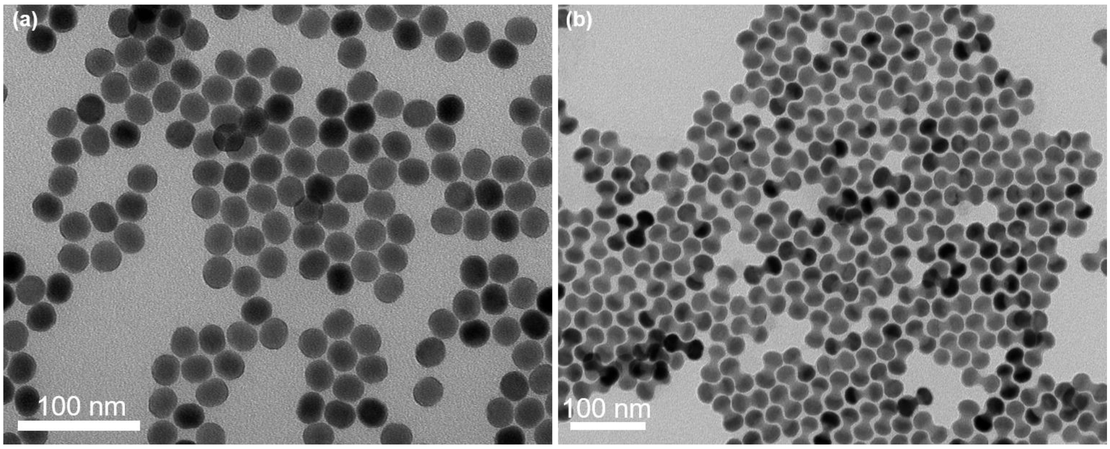

Figure S1. TEM images of β-$NaYF_4$:Yb/Er core (a) and $NaYF_4$:Yb/Er@$NaGdF_4$ (b) core-shell NDs, respectively.

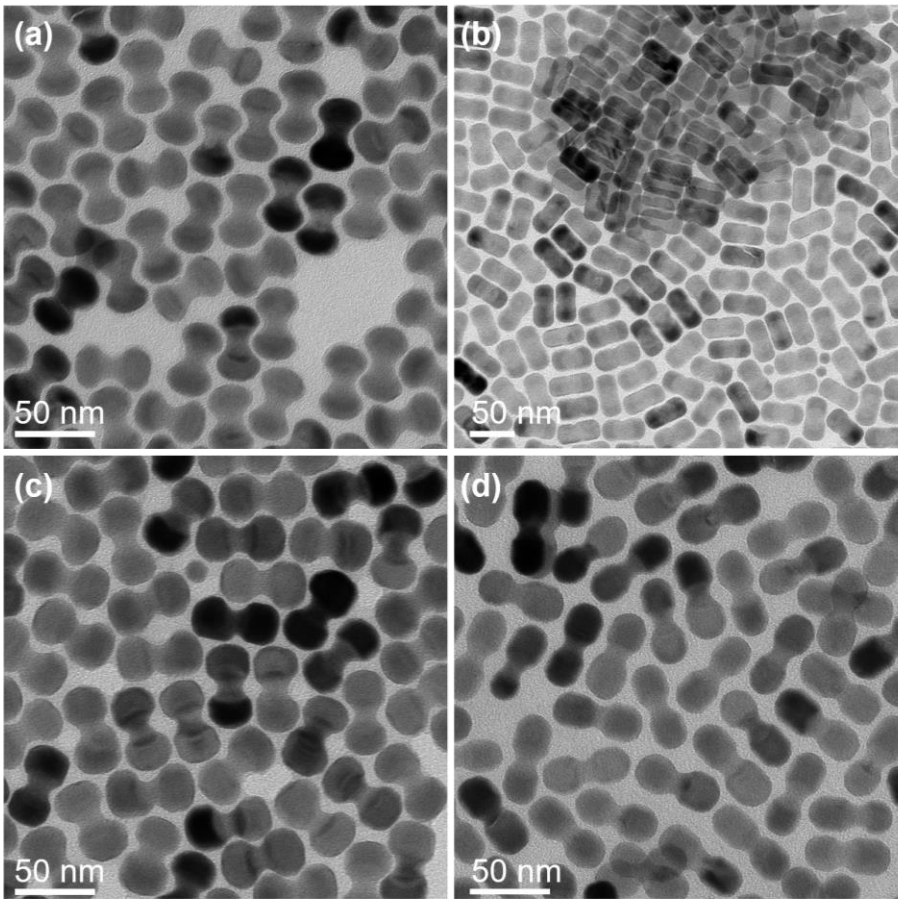

Figure S2. Characterizations of NDs with different aspect ratios. (a)-(d) TEM images of different samples with aspect ratios of *L/D*=1.07, 1.08 (the sample with same value of *L/D* can be differentiated by *D/d*=1.09), 1.18, 1.48, respectively.

Table S1. The shape parameters of different ND samples.

Sample	*L* (nm)	*D* (nm)	*d* (nm)	*L/D*	*D/d*
a	27	25	12	1.08	2.08
b	29	27	12	1.07	2.25
c	26	24	22	1.08	1.09
d	33	28	15	1.18	1.87
e	34	23	13	1.48	1.77

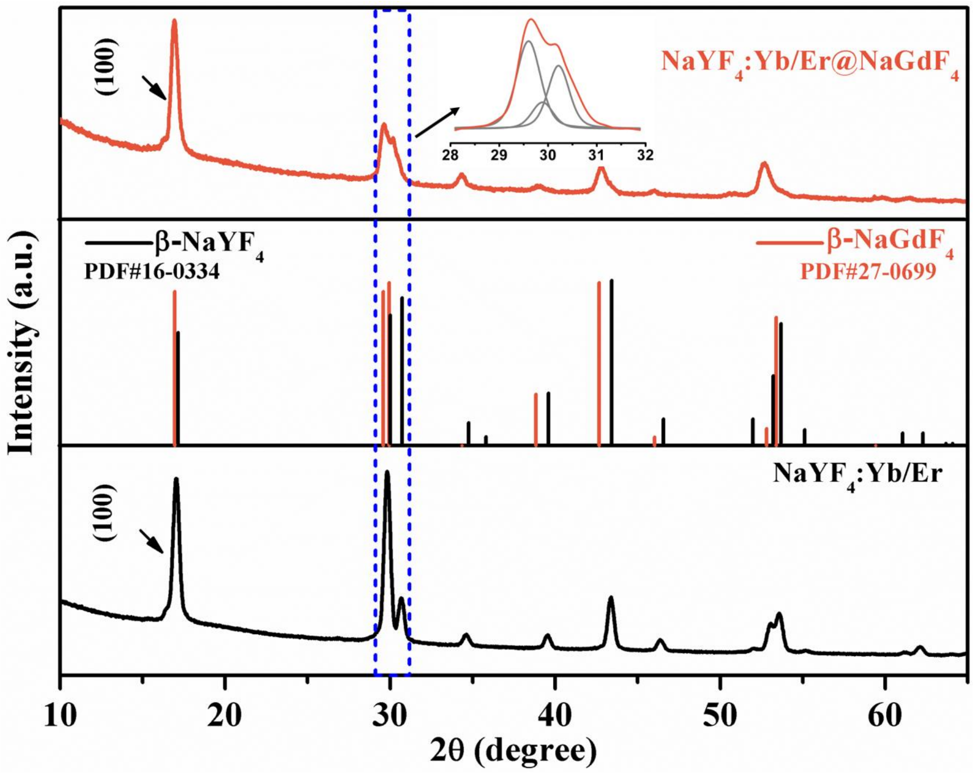

Figure S3. XRD results of β-$NaYF_4$:Yb/Er core and $NaYF_4$:Yb/Er@$NaGdF_4$ core-shell NDs.

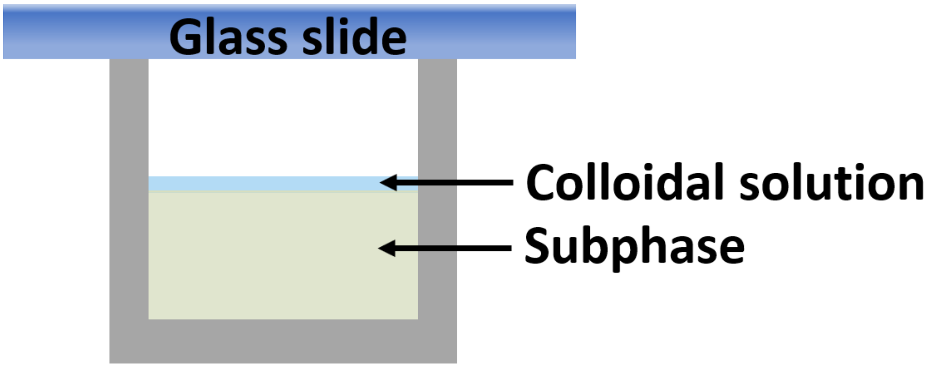

Figure S4. Schematic of the self-assembly experiment at the liquid-air interface.

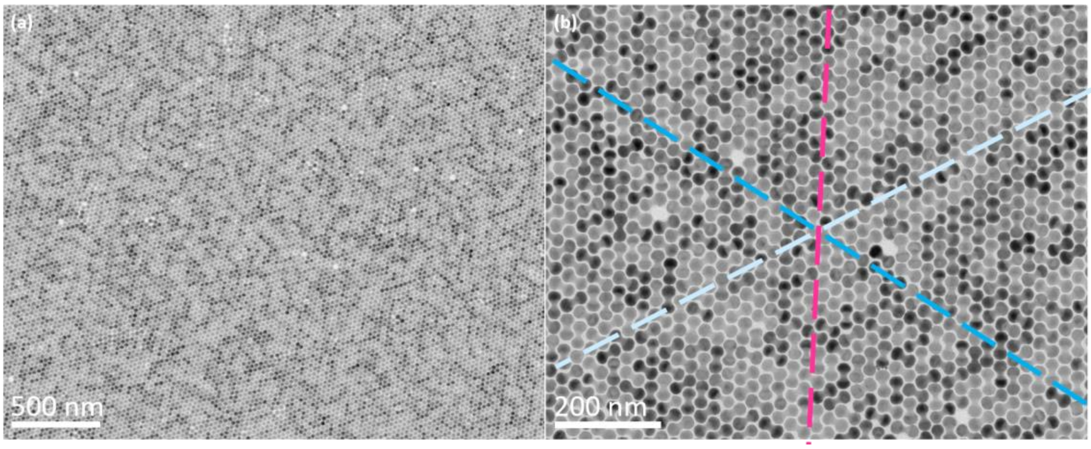

Figure S5. (a)-(b) TEM images of monolayer membrane of NDs self-assembled on EG subphase at different magnifications. Three dashed lines with different color in Figure S5b represents three different ND orientations.

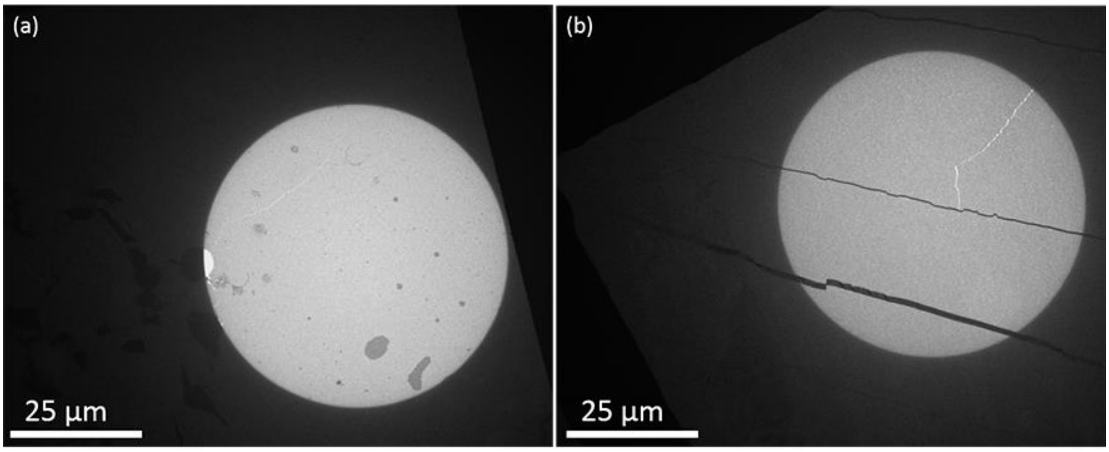

Figure S6. TEM images of large-scale monolayer membrane (a) and bilayer membrane (b) self-assembled on EG subphase.

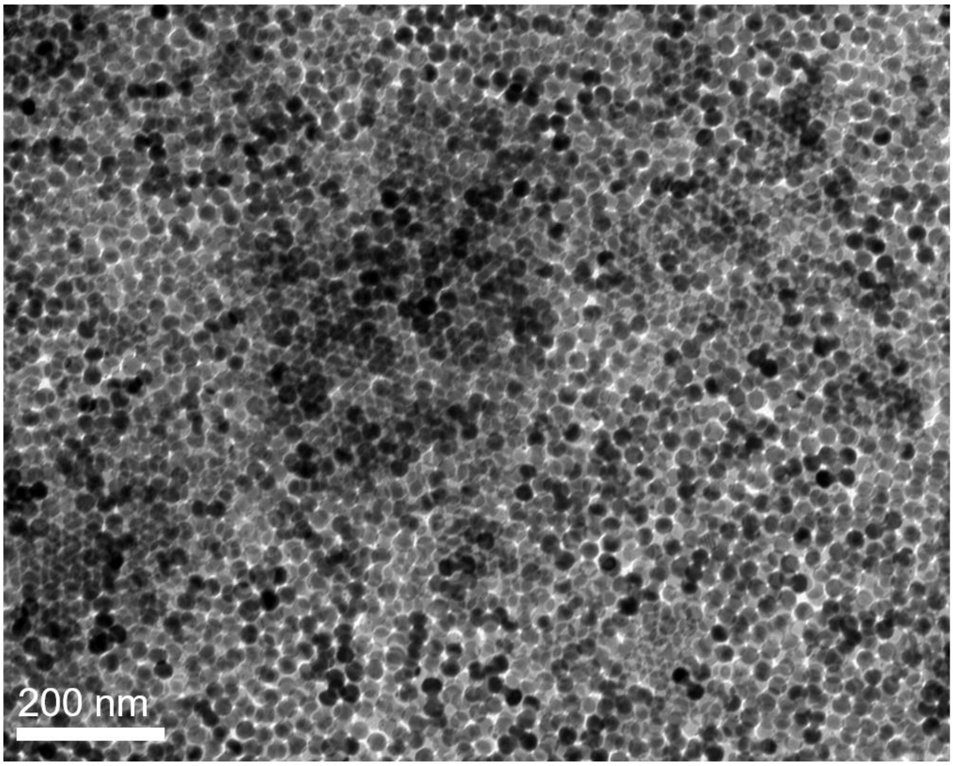

Figure S7. TEM image of films of NDs self-assembled on EG subphase without adding toluene co-solvent to the stock solution.

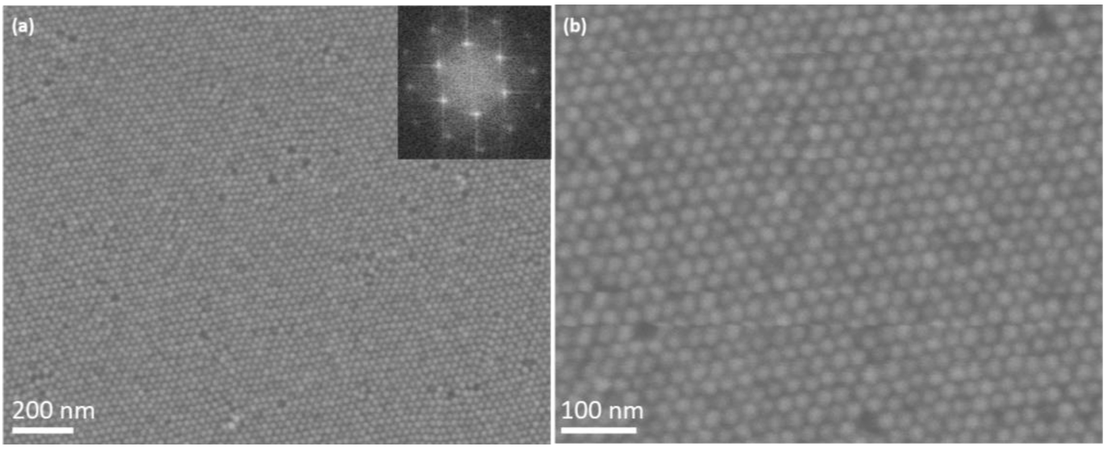

Figure S8. (a)-(b) SEM images of NDs self-assembled on EG subphase at different magnifications. Inset of (a) shows the corresponding FFT pattern.

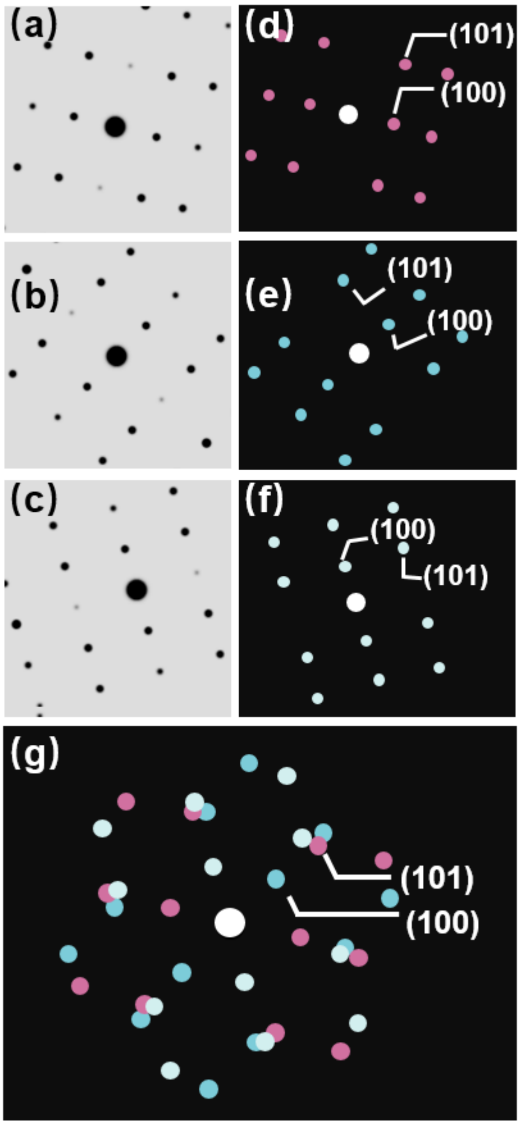

Figure S9. Simulated FFT patterns of NDs with three different orientations. (d-f) show the FFT patterns labeled with three different colors corresponding to (a-c), respectively. (g) Simulated FFT pattern obtained from the combination of FFT patterns in (d-f).

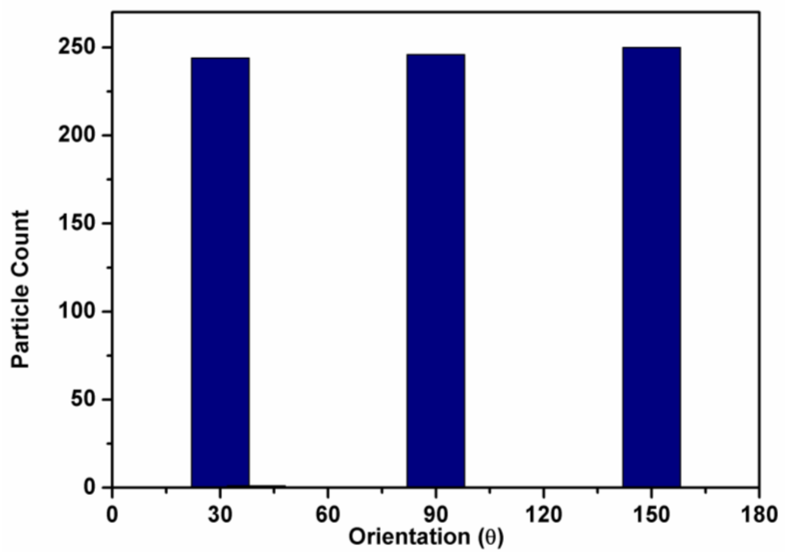

Figure S10. Histogram of particle orientations taken from Figure S10a. The first peak measured was shifted to 30 degrees to illustrate the trimodal distribution clearly.

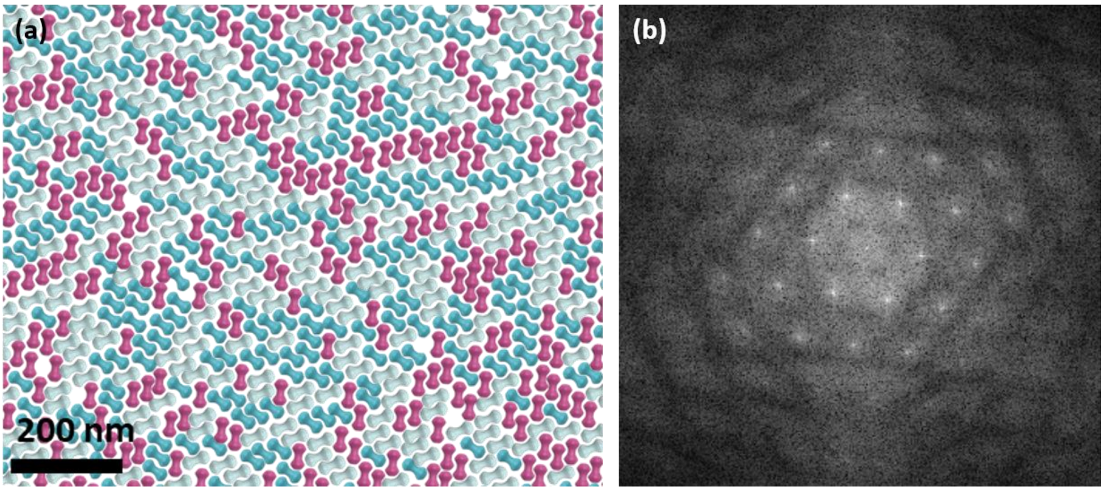

Figure S11. Schematic of monolayer assembly (a) and corresponding FFT pattern (b).

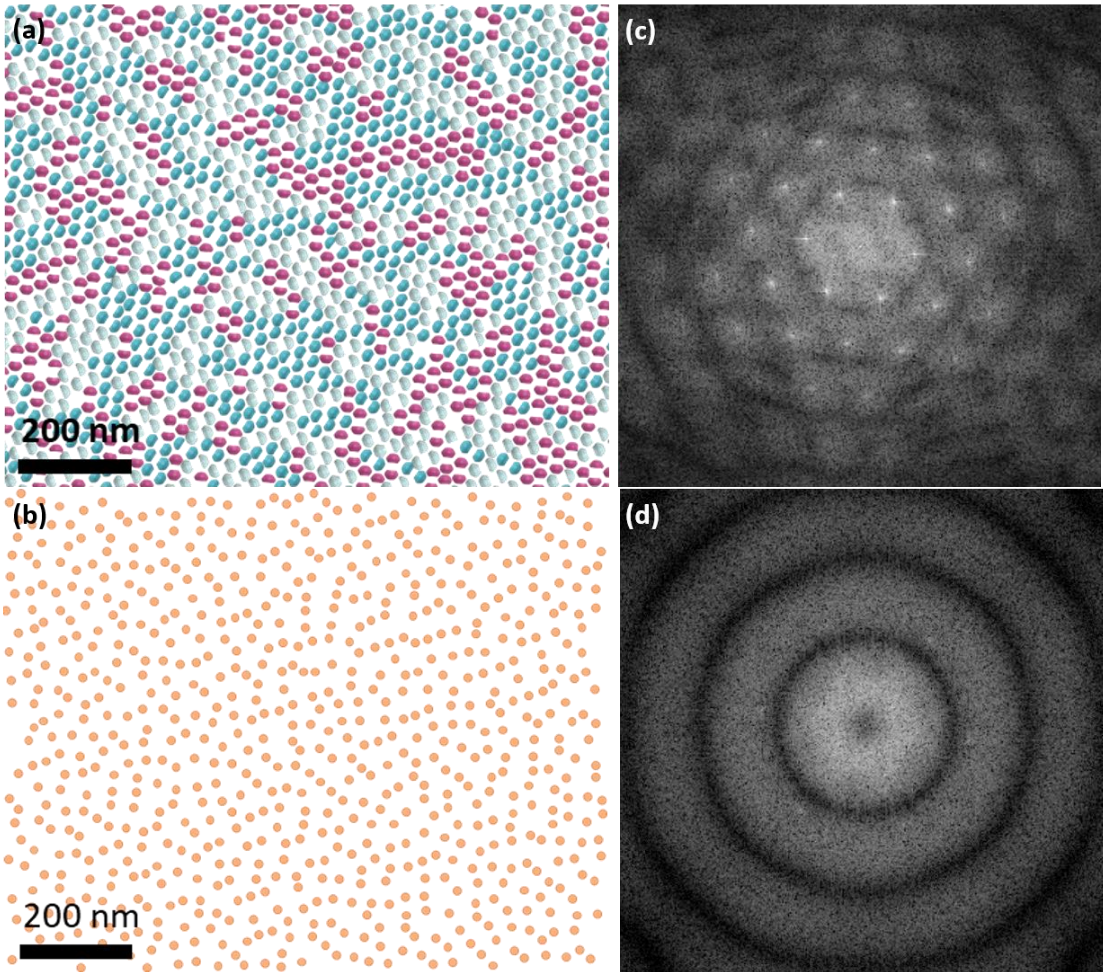

Figure S12. FFT analyses of NDs in monolayer structure. Schematics of separate heads (a) and middle parts (b) of NDs from Figure S8. The middle rod-like parts are replaced with spheres to exclude the effect of irregular shape on FFT analyses. FFT patterns of separate heads (c) and middle parts (d) of NDs based on (a) and (b), respectively.

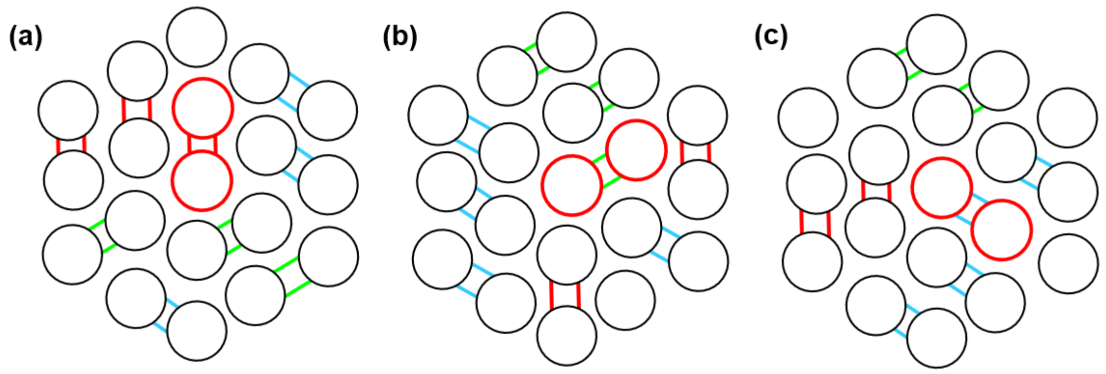

Figure S13. Schematic of hexagonal packing of spheres with lines connecting two neighboring spheres.

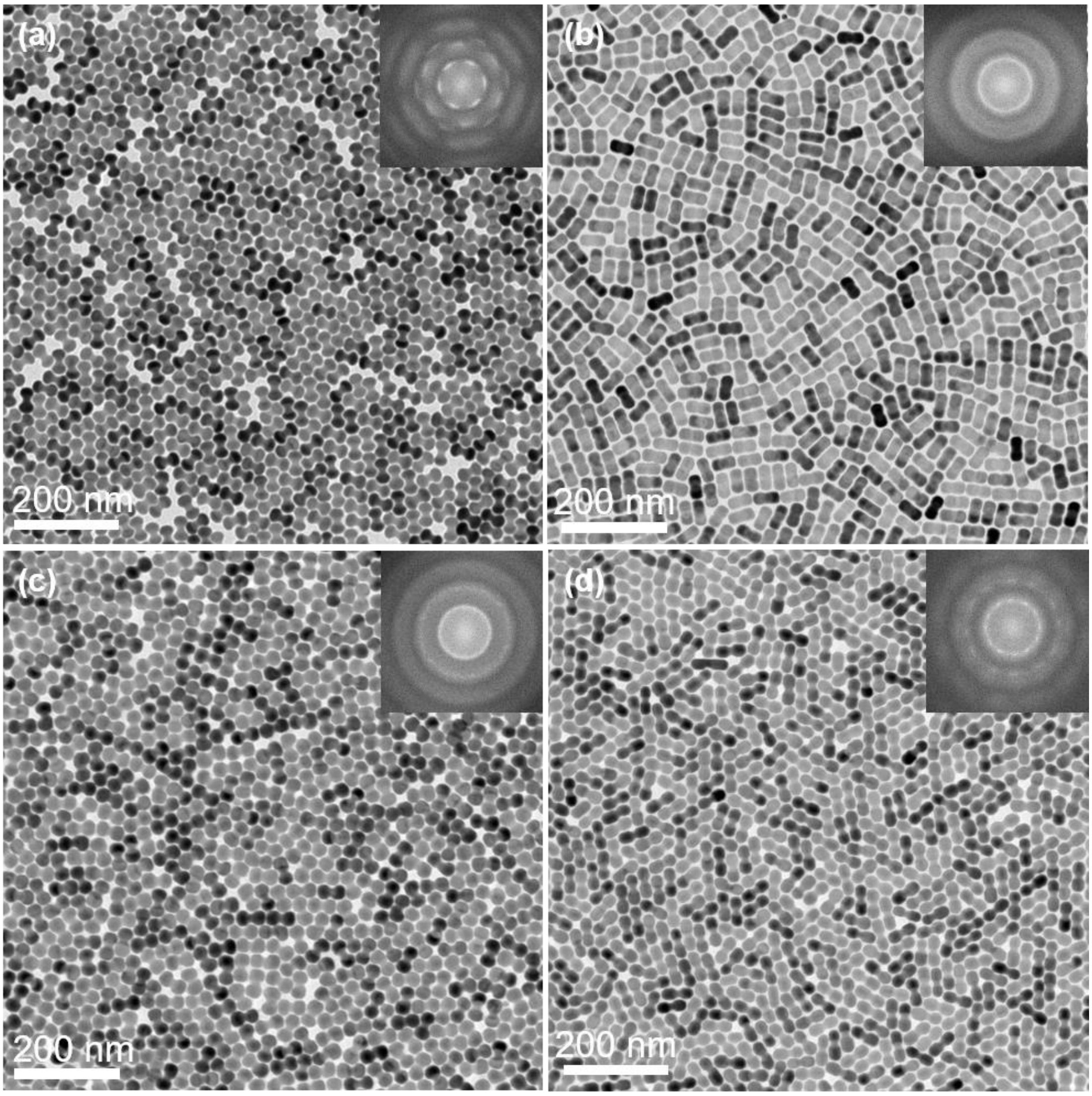

Figure S14. TEM image of NPs with different size aspect ratios self-assembled on EG subphase. (a)-(d) TEM images of NPs with different size aspect ratios self-assembled on EG subphase, corresponding to *L/D*=1.07, 1.08, 1.18, 1.48, respectively. Insets of figures are corresponding FFT patterns.

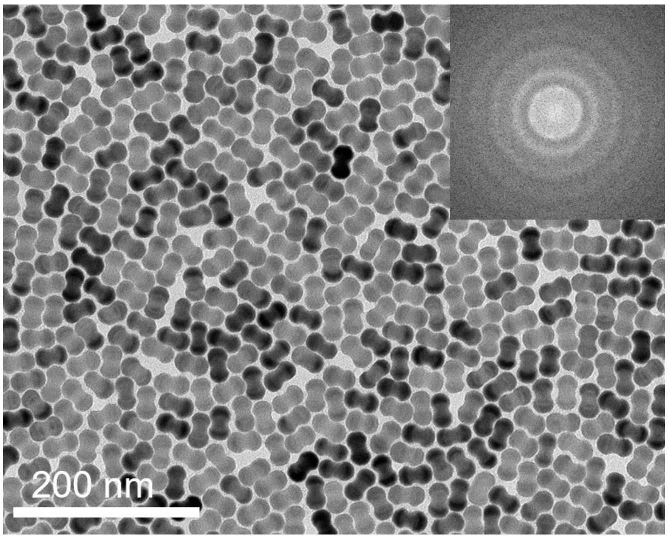

Figure S15. TEM image of NPs with L/D=0.8 self-assembled on EG subphase.

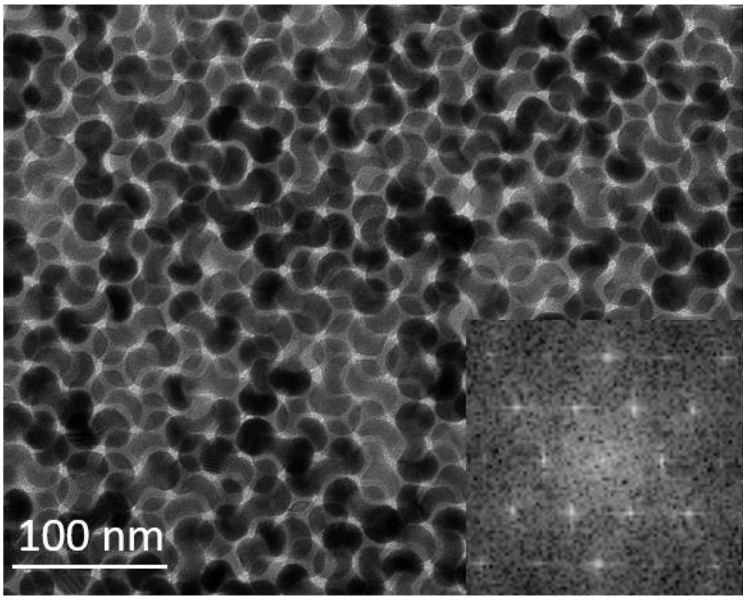

Figure S16. TEM image of bilayer structure with inset showing the corresponding FFT pattern.

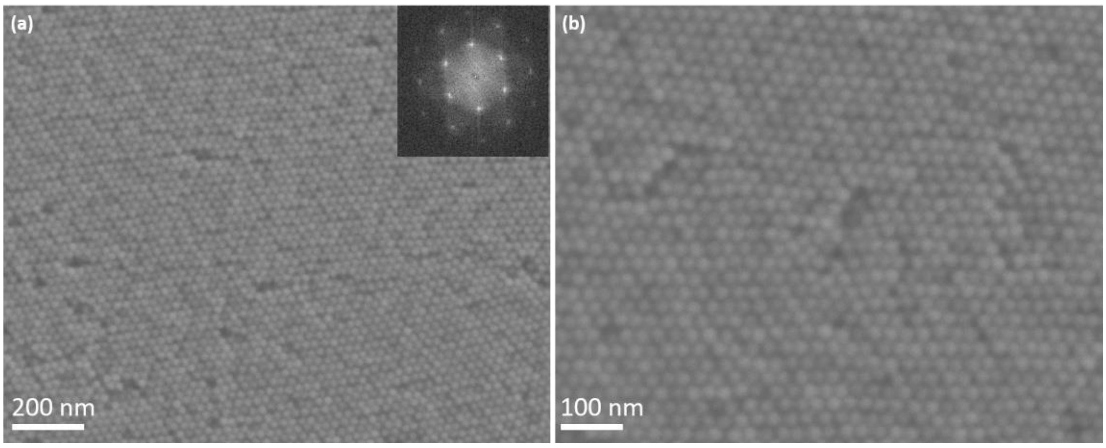

Figure S17. SEM images of bilayer structure at different magnifications. Inset of (a) shows the corresponding FFT pattern.

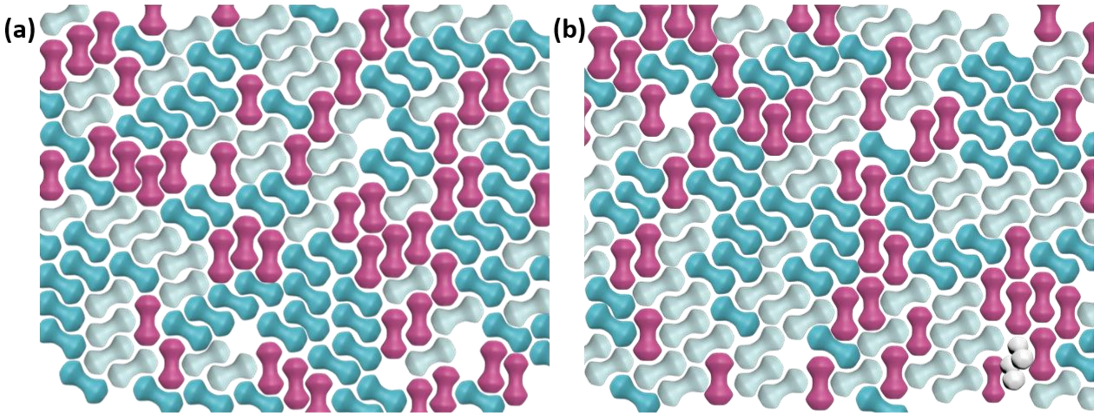

Figure S18. Schematics of the first layer (a) and the second layer (b) captured from the bilayer membrane.

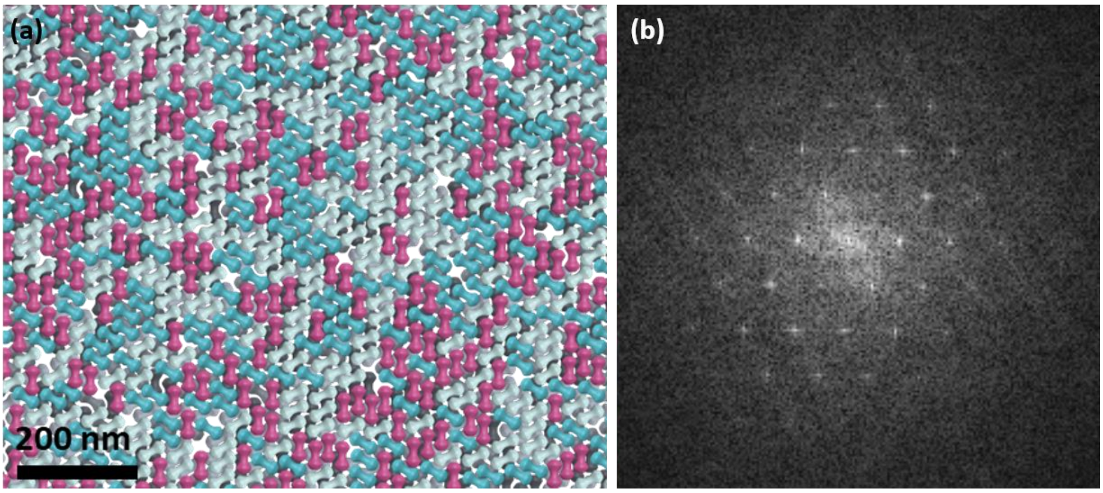

Figure S19. Schematics of monolayer assembly (a) in bilayer superstructure and corresponding FFT pattern (b).

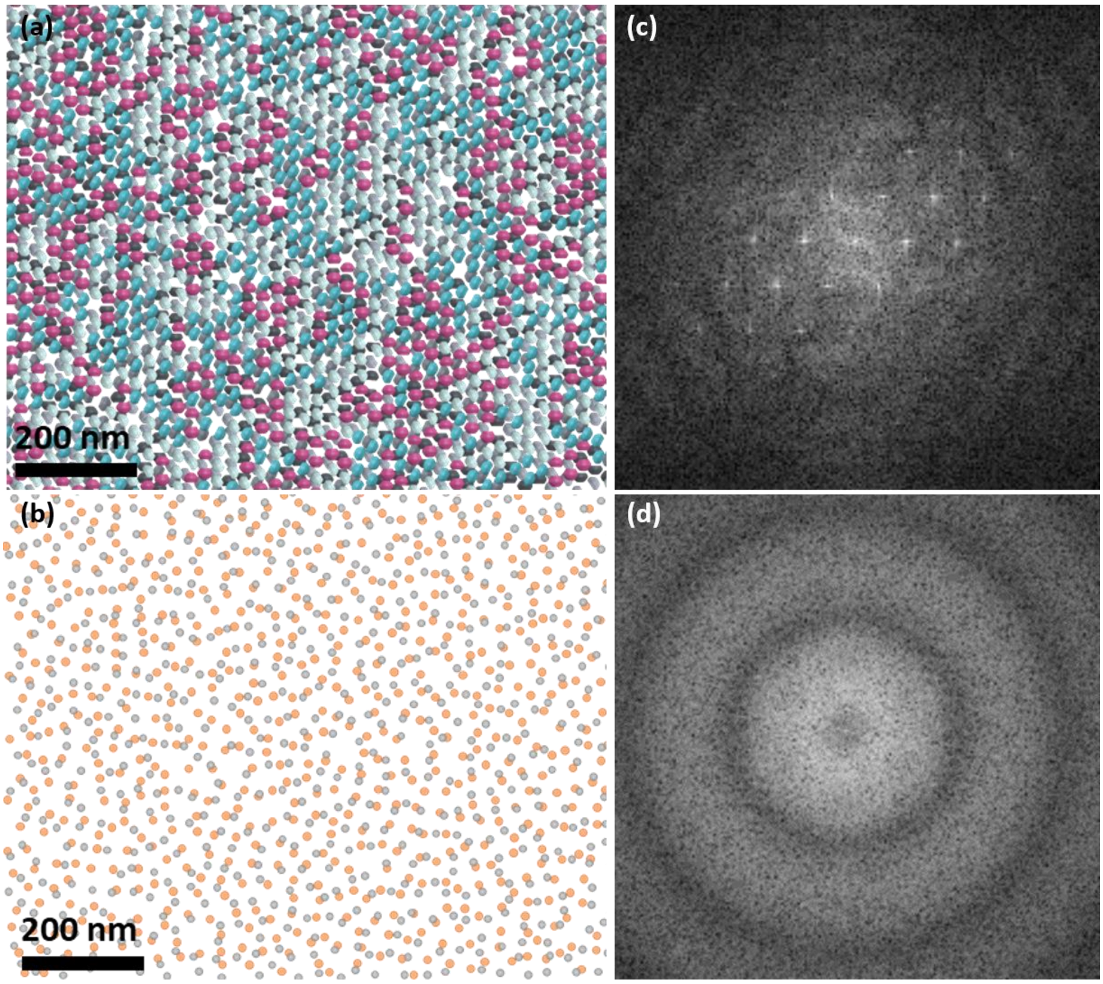

Figure S20. FFT analyses of monolayer in bilayer superstructure. Schematics of separate heads (a) and middle parts (b) of NDs from Figure S15. The middle rod-like parts are replaced with spheres to exclude the effect of irregular shape on FFT analyses. FFT patterns of separate heads (c) and middle parts (d) of NDs based on (a) and (b), respectively.

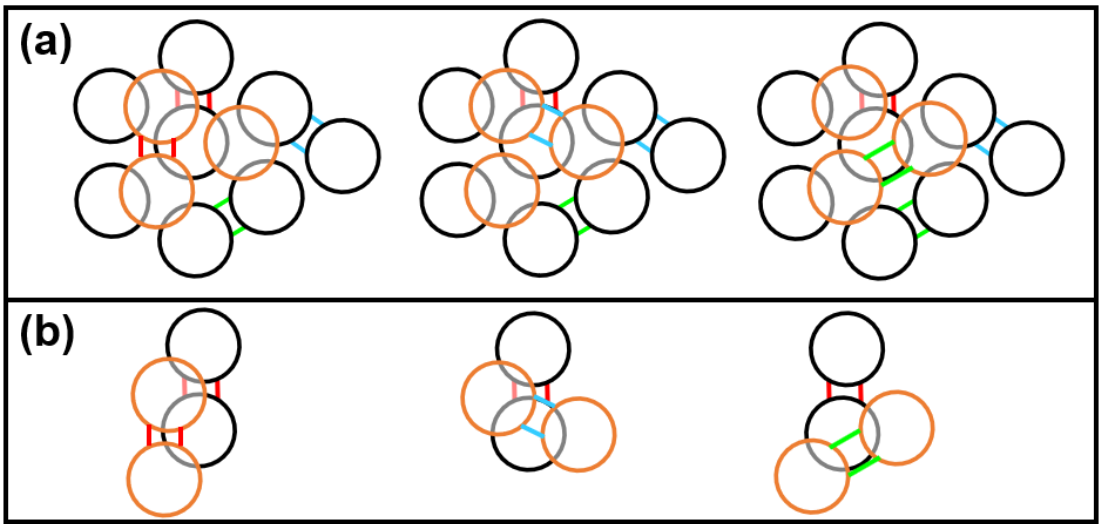

Figure S21. (a) Schematics of double-layer spheres with hexagonal packing of AB stacking, in which three different ways of connecting two neighboring spheres in the same layer are presented. Black spheres represent the NDs in the first layer, and the orange ones represent the NDs in the second layer. (b) Simplified schematics extracted from (a).

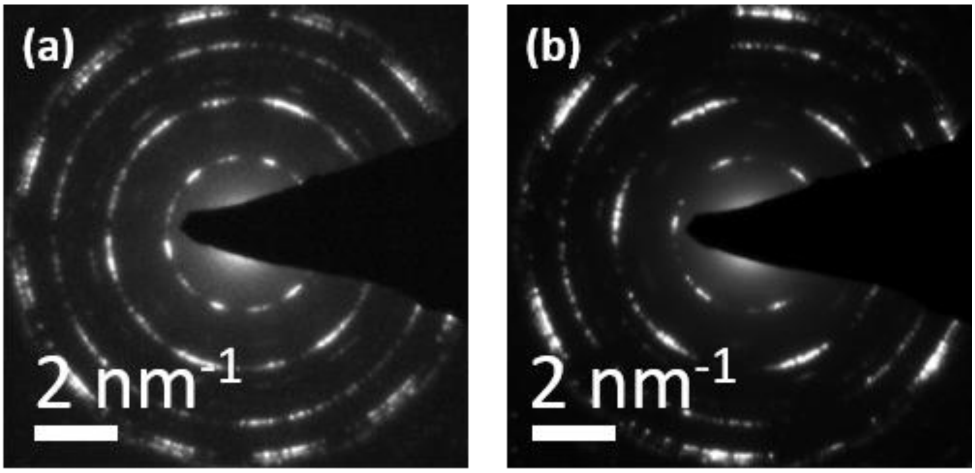

Figure S22. SAED patterns of two different moiré patterns with relative rotations of 30 degrees (a) and 18 degrees (b) between two layers, respectively.

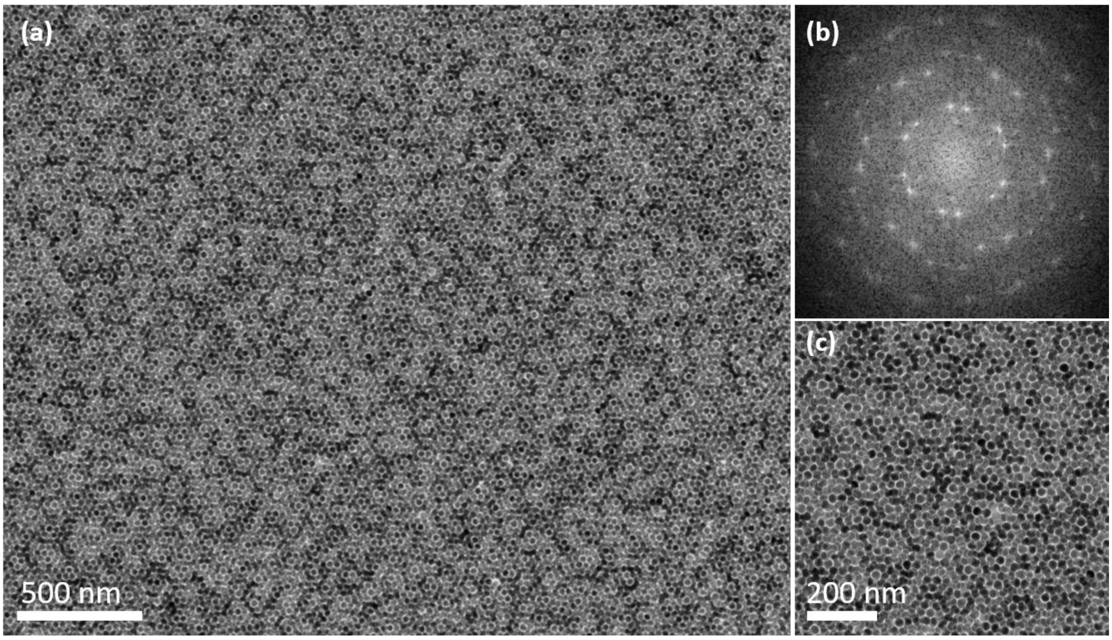

Figure S23. Moiré pattern formed from two layers with the relative rotation of 18 degrees. (a) TEM image of large-scale moiré pattern. (b) Corresponding FFT pattern. (c) TEM image at a higher magnification.

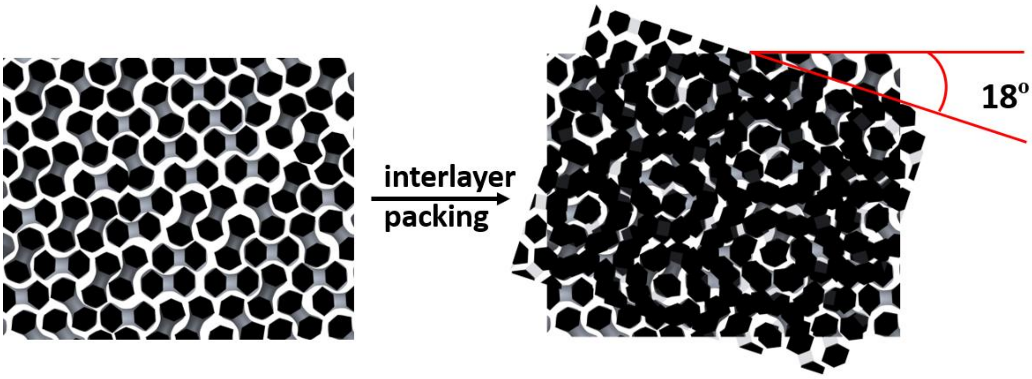

Figure S24. Schematic of the formation of Moiré pattern with the rotation angle of 18 degrees.

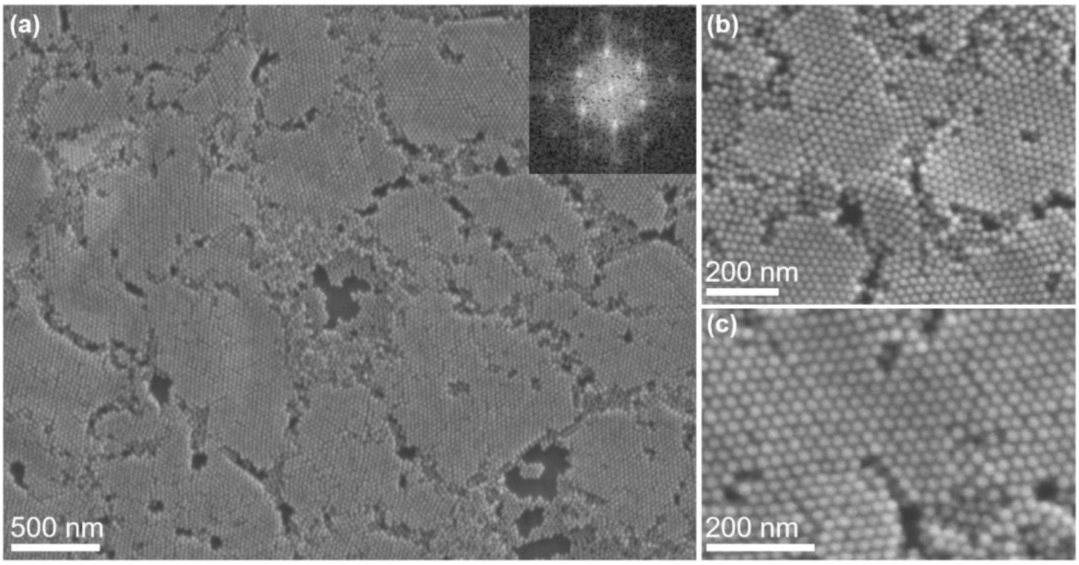

Figure S25. SEM images of films of NDs (*L/D*=1.08, *l/d*=1.42) self-assembled on DMSO subphase at different magnifications. (a-c) SEM images of tilted NDs at different magnifications. Inset of (a) shows corresponding FFT pattern.

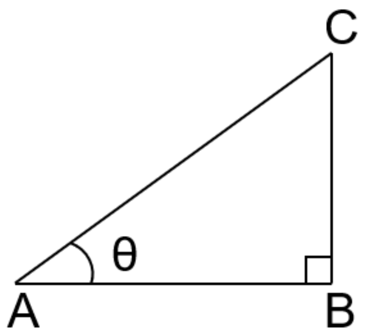

Figure S26. Simplified figure of a triangle, in which line “AB” represents the length of projection of tilted ND in the horizontal direction and can be measured from TEM image of tilted NDs. Line “AC” represents the length of ND, which can be measured from the TEM image of horizontal NDs.

$\cos\theta = L_{AB}/L_{AC} = 40/50 = 0.8$

$\theta = \arccos\theta = 36\,^{o}$

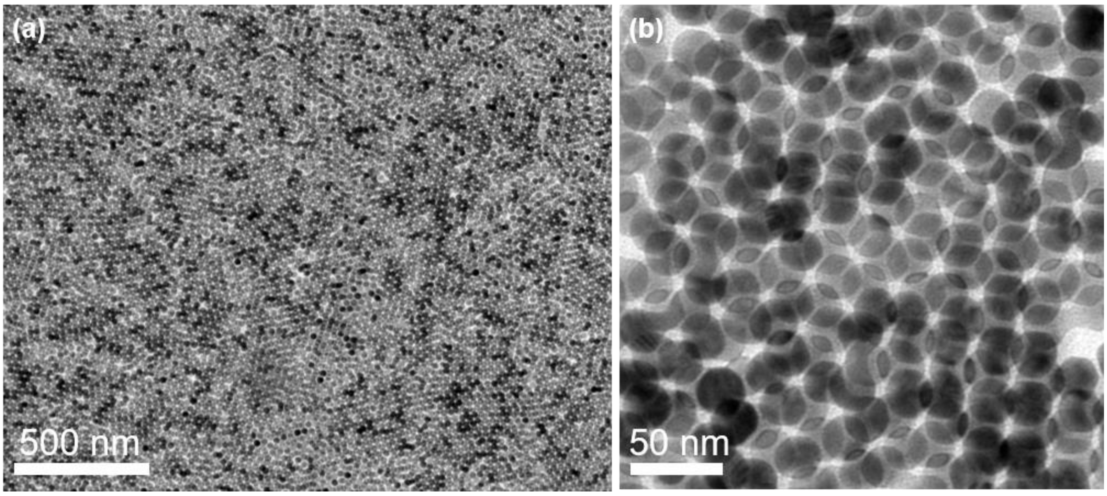

Figure S27. NDs (*L/D*=1.08, *l/d*=1.42) self-assembled at DMSO subphase with only hexane used as solvent.
